# Mechanisms of esophageal cancer metastasis and treatment progress

**DOI:** 10.3389/fimmu.2023.1206504

**Published:** 2023-06-08

**Authors:** Yusheng Wang, Wei Yang, Qianyun Wang, Yong Zhou

**Affiliations:** ^1^ Department of Thoracic Surgery, The First People’s Hospital of Changzhou, Changzhou, Jiangsu, China; ^2^ Department of Gastroenterology, Kunshan Hospital of Traditional Chinese Medicine, Kunshan, Jiangsu, China

**Keywords:** metastasis, esophageal cancer, anatomical mechanism, molecular mechanism, chemotherapy, immunotherapy, targeted therapy

## Abstract

Esophageal cancer is a prevalent tumor of the digestive tract worldwide. The detection rate of early-stage esophageal cancer is very low, and most patients are diagnosed with metastasis. Metastasis of esophageal cancer mainly includes direct diffusion metastasis, hematogenous metastasis, and lymphatic metastasis. This article reviews the metabolic process of esophageal cancer metastasis and the mechanisms by which M2 macrophages, CAF, regulatory T cells, and their released cytokines, including chemokines, interleukins, and growth factors, form an immune barrier to the anti-tumor immune response mediated by CD8+ T cells, impeding their ability to kill tumor cells during tumor immune escape. The effect of Ferroptosis on the metastasis of esophageal cancer is briefly mentioned. Moreover, the paper also summarizes common drugs and research directions in chemotherapy, immunotherapy, and targeted therapy for advanced metastatic esophageal cancer. This review aims to serve as a foundation for further investigations into the mechanism and management of esophageal cancer metastasis.

## 1 Introduction

Esophageal cancer is a common malignant tumor of the upper gastrointestinal tract and is currently ranked seventh in terms of incidence and sixth in terms of mortality worldwide (1). Due to the lack of obvious early clinical symptoms, most patients are diagnosed with esophageal cancer at the middle and late stages, often with distant metastases (2). Primary esophageal cancer is histologically divided into two types: esophageal squamous cell carcinoma (ESCC) and esophageal adenocarcinoma (EAC), with each type having different types of metastases occurring at different stages. Stages 1a and 1b, with an invasion depth of sm1, are generally considered to be free of lymph node metastasis, while the chances of lymph node metastasis increase continuously thereafter (3).

The metastasis of esophageal cancer can be classified into lymphatic metastasis, hematogenous metastasis, and direct diffusion metastasis. Direct diffusion metastasis usually occurs later when the tumor invades adjacent tissues after penetrating the loose outer membrane. Hematogenous metastasis mostly occurs after lymph node metastasis, spreading to distant organs through blood vessels. Recent clinical studies have reported pancreatic metastases from esophageal squamous cell carcinoma to the lung, pleura, liver, stomach, kidney, and pancreas (4), as well as skeletal muscle metastases (5) and distant metastases in the thyroid gland (6). Lymphatic metastasis is the main mechanism of esophageal cancer metastasis and a significant factor influencing its prognosis (7, 8). The 7th edition of postoperative TNM staging for esophageal cancer, jointly developed by JACC and UICC, classifies lymph node metastases based on the number of lymph node metastases into four stages: N0, N1, N2, and N3. N0 indicates no lymph node metastasis, while N1, N2, and N3 indicate 1-2, 3-6, and 7 or more lymph node metastases, respectively (9). Apart from general lymphatic metastasis, esophageal cancer can also exhibit lymphatic jump metastasis (NSM). Different institutions have varying definitions of NSM; for instance, the Japanese Esophageal Society (JES) assigns numbers and names to lymph nodes in the neck, mediastinum, and abdominal cavity, dividing them into four substations (1, 2, 3, and 4) based on their location relative to the primary tumor. NSM is defined as the absence of metastasis in lymph nodes at station 1 and the presence of one or more metastases in lymph nodes at stations 2, 3, and 4 (10, 11). The American Cancer Society (AJCC) has a similar definition of NSM, but the difference lies in the definition of station 1 lymph nodes. The AJCC classification simplifies it by considering only paraesophageal lymph nodes as station 1, whereas the JES has a more detailed classification, dividing station 1 lymph nodes into three segments in the upper, middle, and lower thorax (12, 13).

The process of esophageal cancer metastasis involves various molecular mechanisms, encompassing a wide array of cytokines, specific proteins, and tumor-associated cells that collectively constitute the tumor metastasis microenvironment. Cytokines, such as inflammatory factors, chemokines, and growth factors, play a crucial role in tumor growth and invasion. For instance, VEGF and EGF can stimulate angiogenesis and lymphangiogenesis. CC chemokines can induce immune cell migration to the tumor microenvironment, promoting tumor progression. Inflammatory factors like IL can activate Treg and other immune cells, triggering the release of additional cytokines and participating in angiogenesis, tumor migration, and immunosuppression. Special proteins like MMP, ITG, and cadherin are involved in ECM degradation and tumor extravasation by disrupting the extracellular matrix and enhancing intercellular adhesion. Tumor-associated cells, particularly immune cells, differentiate into phenotypes conducive to tumor cell protection from cytotoxic immune cell destruction through immunosuppression, driven by the cytokines secreted by tumor cells.

The majority of esophageal cancer patients are diagnosed with metastasis, where the significance of multidisciplinary comprehensive treatments such as chemotherapy, immunotherapy, and targeted therapy surpasses that of surgical treatment. The final section of this paper provides a review of medications, their influencing factors, and the use of chemotherapy and immunotherapy. Additionally, some potentially beneficial targets are introduced, with the hope of providing a foundation for future research.

## 2 Mechanism of esophageal cancer metastasis

### 2.1 Anatomical mechanisms of metastasis

The esophagus is anatomically divided, from inner to outer layers, into the mucosal layer, submucosal tissue, intrinsic muscle layer (consisting of circumferential and longitudinal muscle layers), and outer membrane. In cases where esophageal cancer infiltrates from the outer membrane, it can spread to surrounding tissues and involve the pleura, pericardium, trachea, and other adjacent structures (14).

The arterial supply to the esophagus primarily occurs in the mucosal and submucosal layers and can be categorized into cervical, thoracic, and abdominal segments based on location. The cervical arterial supply largely originates from the subxiphoid artery. The thoracic arterial supply mainly arises directly from the aorta and bronchial artery, with a smaller portion originating from the intercostal artery. The abdominal arterial supply branches from the left subphrenic artery, left gastric artery, and short gastric artery. Esophageal veins typically accompany the arteries (15). Metastasis to other organs through the bloodstream can occur, such as to the stomach via the gastric artery or to the bronchi and lungs via the bronchial artery. However, the precise anatomical mechanisms of hematogenous metastasis are still being investigated.

The lymphatic vessels in the esophagus are primarily located in the submucosal layer, muscularis mucosae, and intrinsic muscularis layer. Lymphatic vessels in the submucosal and muscularis layers predominantly extend longitudinally along the esophagus, facilitating longitudinal drainage of lymphatic fluid (16, 17). Conversely, intermuscular lymphatic vessels in the intrinsic muscularis layer tend to extend in a circular fashion, contributing to circular drainage at that level. The thoracic duct, extraesophageal lymphatics, and lymphatic vessels in the submucosal and mucosal muscle layers are connected to the right laryngeal recurrent nerve lymph node and the inferior ramus lymph node. They can also directly connect to the thoracic duct after transferring tumor cells to systemic lymph nodes and to the extraesophageal lymphatics through intermuscular lymphatic flow (18, 19). Through these pathways, tumor cells can metastasize to lymph nodes in the neck, mediastinum, paratracheal, paraesophageal, abdomen, and other regions. Paratracheal, paraesophageal, perigastric, right laryngeal nerve lymph nodes, and inferior ramus lymph nodes are commonly associated with lymph node metastasis (20). The anatomical pattern of lymph node metastasis also determines the extent of surgical clearance. A study in 2023 comparing lymph node metastasis patterns between adenocarcinoma of the esophagogastric junction (AEG) and lower thoracic esophageal squamous cell carcinoma (ESCC) found that ESCC was more likely to invade the lower mediastinal and paracardial lymph nodes than AEG. The presence of lymph node metastasis in these regions in AEG indicates a stage T3 or higher, necessitating complete lymph node clearance of the lower mediastinum and abdomen in advanced AEG (21).

When tumor cells penetrate the esophageal wall through longitudinal drainage ducts and subsequently traverse transverse drainage ducts, there is a higher possibility of bypassing lymph nodes near the primary tumor and metastasizing to more distant lymph nodes, promoting the development of lymph node skip metastasis (22, 23). Furthermore, a Japanese study found that lymphatic skip metastasis in thoracic esophageal carcinoma was associated with the absence of lymphatic vessels between the middle mediastinum and supraclavicular muscles. Generally, metastasis of thoracic esophageal squamous cell carcinoma commonly occurs in the upper and lower mediastinum but rarely in the middle mediastinum. Middle mediastinal lymph node metastases are more likely to be jump metastases through direct invasion of lymphatic vessels or lymph nodes in the internal and external mediastinal muscles. Lymph node jump metastases can sometimes provide a basis for subsequent treatment. In a study in 2023, lymph node jump metastases after mesothoracic ESCC were found to be a valid prognostic indicator for ESCC patients. Additionally, patients in the postoperative NSM (lymph node skip metastasis) group were found to be more responsive to chemoradiotherapy (24).

### 2.2 Tumor metabolism and immunosuppression affect T cell processes

Given the limited number of published papers on the molecular mechanisms of direct diffusion metastasis, this section primarily focuses on elucidating the molecular mechanisms underlying hematogenous and lymphatic metastasis.

#### 2.2.1 Tumor metabolism and related substances during hematogenous

##### 2.2.1.1 Premetastatic niche formation

**Figure 1:**
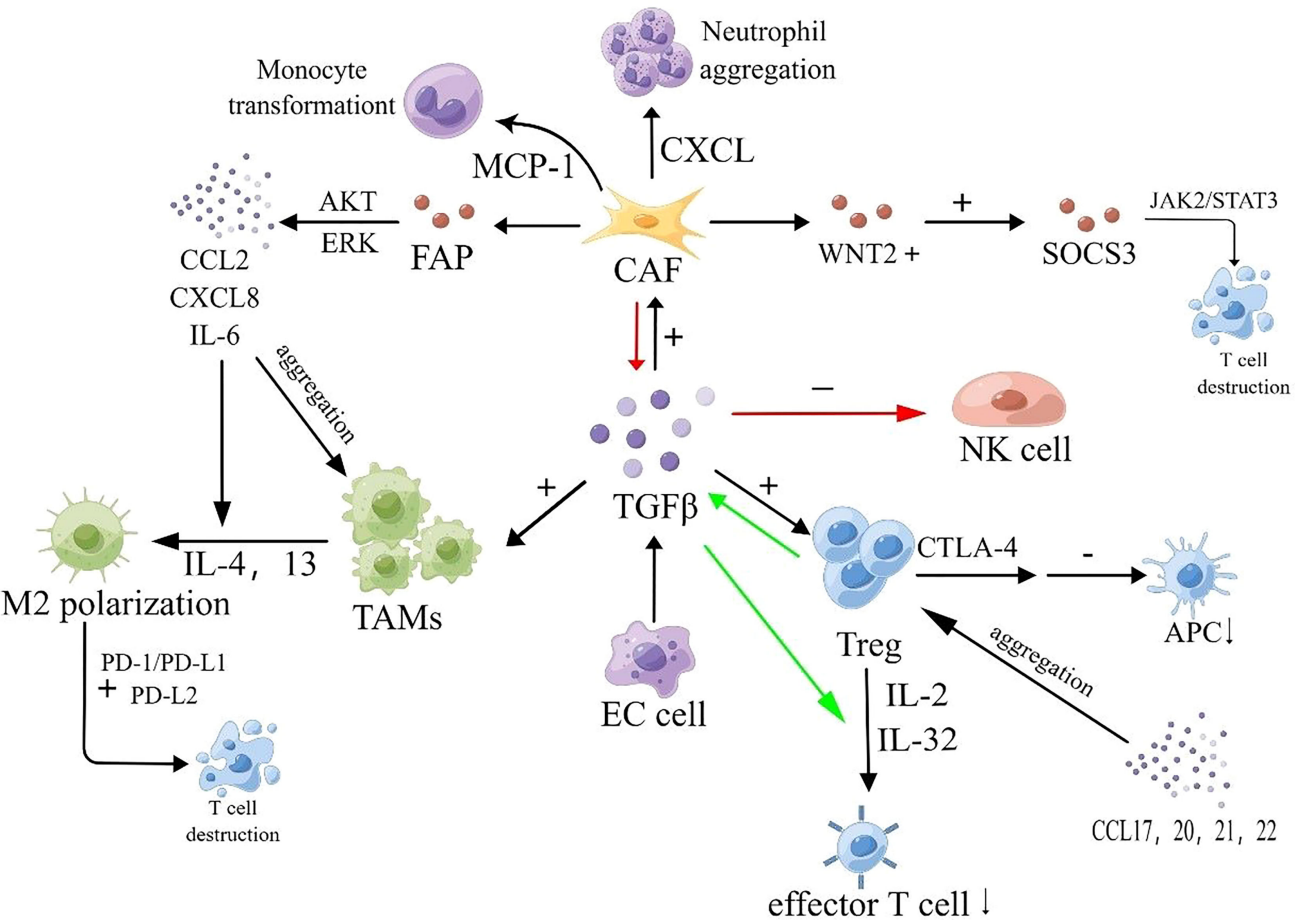
Hematogenous transfer process. VEGF-A and VEGF-B bind to VEGFR-1 and VEGFR-2, initiating downstream signaling pathways that promote angiogenesis. Tumor-derived exosomes can enhance angiogenesis and facilitate the release and transport of MMPs, influencing angiogenesis. TGF-β1 released by tumors activates smad2/3 and inhibits smad7, leading to the production of N-calmodulin and Vimentin, and suppressing the expression of E-calmodulin, thereby inducing and accelerating the process of EMT. MMPs contribute to ECM disruption. Tumor cells enhance adhesion to the basement membrane through ITGα1 and E-calmodulin. Members of the ITGαβ family facilitate EC cell transmigration across the vascular endothelium. P-selectin interacts with talin1 to activate integrin αIIbβ3, which recruits platelets to peri-CTCs. P-selectin, fibronectin (FN), and platelet granules (PG) promote CTC clustering by inducing platelet aggregation around CTCs. Platelet adhesion and aggregation trigger the secretion of p-selectin and TGF-β1 via the TGF-β1-garp pathway, leading to increased FN synthesis. This provides mechanical protection against shear forces and immune cell attacks. Additionally, fibrin binding to leukocytes contributes to the formation of small clots, further enhancing the stability of CTC clusters.

The molecular mechanisms of hematogenous metastasis in various cancers share similarities, and the same mechanisms can be attributed to hematogenous metastasis in esophageal cancer. During the premetastatic stage, tumor cells create a premetastatic environment in distal tumors with the assistance of various substances, including growth factors and chemokines secreted by cancer cells or stromal cells. For instance, vascular endothelial growth factor (VEGF) secreted by cancer cells affects calmodulin junctions in vascular endothelium, leading to increased vascular permeability. VEGF-A and VEGF-B bind to VEGFR-1 and VEGFR-2, transmitting signals and activating downstream signaling pathways to promote angiogenesis. Tumor vessels, formed as a result, indirectly aid tumor cell entry into the circulation (25–27). The Pi3k/Akt signaling pathway has been identified as a major downstream pathway mediating the biological actions of VEGF-A, and the knockdown of VEGF-A has been shown to affect the invasive ability of ECA109 cells (28). Other substances can also influence tumors by modulating the level of VEGF. For example, ANXA2 protein has been found to affect VEGF activity by activating MYC production (29). Chemokines in the tumor microenvironment also play a role in hematogenous metastasis. CXC chemokines, for example, can participate in tumor blood vessel formation (30). Studies have revealed that CXCL12 is abnormally elevated in the serum of esophageal cancer patients, particularly those with poorly differentiated tumors. Based on these findings, it is inferred that the expression of CXCL12 promotes hematogenous and lymphatic metastasis in esophageal cancer, although the specific mechanism requires further exploration (31). Additionally, increased expression of CXCL8 has been observed in esophageal cancer. Immunohistochemical analysis has shown that elevated expression of CXCL-8 and its receptor CXCR-2 is closely associated with invasion depth, pathological stage, and venous invasion in esophageal cancer (32). CC chemokines also play a significant role in the metastasis of esophageal cancer. However, CC chemokines contribute to the immune escape process by attracting Tregs, Th17 cells, and other tumor-related immune cells, which will be discussed later. Inflammatory cytokines, in addition to growth factors and chemokines, also play a crucial role in esophageal cancer metastasis. Increased COX-2 secretion in esophageal squamous cell carcinoma (ESCC) promotes enhanced vascular permeability, thereby accelerating hematogenous metastasis (33). Interleukin (IL) and tumor necrosis factor-alpha (TNF-α) also play significant roles in hematogenous metastasis. IL-1, particularly IL-1β, can induce tumor angiogenesis by directly stimulating mature epithelial cells and promoting the production of pro-angiogenic factors, including VEGF. The main mechanism involves IL-1 binding to the NF-κB binding site on the NFAT gene promoter and stimulating epithelial cells to release growth factors via the SCR-dependent pathway (34). IL-1β can also inhibit the expression of E-cadherin, increase the expression of vimentin, and promote epithelial-mesenchymal transition in esophageal cancer cells (35). IL-32 has been found to stimulate the production of TGF-α through the activation of NF-κB and p-p38 MAPK pathways. TGF-α, in turn, promotes angiogenesis, influencing the process of hematogenous metastasis. TNF-α can induce the expression of MMP-9, which plays a crucial role in angiogenesis, matrix destruction, and cell migration. Furthermore, TNF-α can induce the Warburg effect, increasing aerobic glycolysis, ATP production, lactate secretion, and tumor growth promotion (36) (**Figure 1**).

In addition to growth factors and inflammatory factors, specific proteins are involved in the metastasis of esophageal cancer. One example is matrix metalloproteinases (MMPs), a class of zinc-containing peptidases that play a role in tissue remodeling and wound healing in the body’s microenvironment (37). MMPs can disrupt tight junctions between cells by degrading the extracellular matrix (ECM), leading to proteolysis of the microvascular basement membrane and creating conditions for tumor cell migration (38). MMPs can also release VEGF molecules trapped in the tumor matrix, influencing the angiogenesis process (39). MMPs promote the recruitment of bone marrow-derived cells into the tumor matrix by mediating the release of KIT ligands, activating VEGF-related genes, and facilitating VEGF/VEGFR binding and signal transduction. Studies have shown that CD13-positive bone marrow cells can affect pericyte coverage of tumor-associated blood vessels. MMPs promote the aggregation of bone marrow cells, which may alter pericyte coverage and promote intravascular infiltration of tumor cells. Among the 28 known MMPs, MMP-1, MMP-2, MMP-9, MMP-10, MMP-14, and MMP-21 have been found to be closely associated with the metastasis and invasion of esophageal cancer. MMP-1 is widely expressed in adenocarcinoma and Barrett’s esophagus, while RNF128 promotes the production of MMP-2 by promoting EGFR phosphorylation, leading to metastasis and invasion of ESCC. MMP-9 promotes tumor angiogenesis and accelerates hematogenous metastasis by degrading type IV collagen in tumor tissue and promoting VEGF release, thereby inducing metastasis and invasion of ESCC (40). Interestingly, MMP-2 and MMP-9 can bind to low-density lipoprotein receptor-related protein 1 (LRP1), activate ERK, inhibit the JNK pathway, facilitate tight adhesion of tumor cells to the stroma, and induce tumor cell invasion into blood vessels and lymphatic vessels (41). Reduced expression levels of MMP-14 inhibit infiltration and proliferation of esophageal cancer, suggesting that MMP-14 and MMP-21 can mediate immune evasion of tumor cells through specific regulatory networks. Another important group of MMPs is membrane-type matrix metalloproteinases (MT-MMPs). Increased expression levels of MT1-MMP and MT2-MMP significantly impact the prognosis of esophageal cancer and are considered key factors in promoting its metastasis (42) (**Figure 1**).

Apart from MMPs, extracellular vesicles (EVs) such as exosomes also contribute to the premetastatic tumor microenvironment. Exosomes, known for their involvement in vesicular transport within organisms, have been found to play an important role in tumor metastasis and progression. Exosomes derived from tumor cells in hypoxic and low pH environments can promote angiogenesis, modulate the premetastatic tumor microenvironment, and accelerate extracellular matrix (ECM) deposition (43). Interestingly, a recent study discovered that exosomes derived from esophageal squamous cell carcinoma (ESCC) participate in systemic circulation through transfected expression vectors, but their number does not increase with metastatic progression, and the underlying mechanism for this remains unclear (44). Furthermore, exosomes can integrate into other organs and impact metastasis. MicroRNAs present on the exosome membrane can also influence tumor metastasis through exosome trafficking. For instance, high expression of miR-203 has been associated with an effect on lymph node metastasis (to be explained further in the next section). Exosomal long non-coding RNAs (lncRNAs) can also exhibit functions similar to VEGF. Besides exosomes, other types of EVs also play crucial roles. Tumor-derived EVs can induce the conversion of fibroblasts into myofibroblasts, thereby promoting the release of MMPs and affecting the extracellular matrix. EVs can also stimulate epithelial cells to produce substances that disrupt adhesion between tumor cells, facilitating tumor cell invasion into the bloodstream (45). Notably, cancer vesicles and microvesicles derived from tumors have been found to contain bioactive proteins, including MMPs, which significantly impact the stability of the extracellular matrix and indirectly contribute to tumor progression (46) (**Figure 1**).

##### 2.2.1.2 Adhesion to the basement membrane and invasion of blood vessels

During hematogenous metastasis, the adhesion between tumor cells weakens, causing them to detach from surrounding cells, while the adhesion between tumor cells and the basement membrane increases before the cells disperse and invade the extracellular matrix.

Epithelial-mesenchymal transition (EMT) is a crucial step in the invasion process, during which epithelial cells acquire invasive capabilities. Platelet and fibroblast-released cell adhesion molecules and TGF-β play a vital role in this process (47).

Cell adhesion molecules are transmembrane glycoproteins on cell membranes that mediate attachment between tumor cells and other tissue structures, as well as between tumor cells themselves. Examples of cell adhesion molecule families include calmodulin, integrin, and selectin families.

The reduction in E-cadherin expression leads to the loss of epithelial characteristics in cells, such as cell polarity and attachment to the basement membrane, while acquiring a mesenchymal phenotype characterized by migration, invasion, anti-apoptosis, and extracellular matrix degradation. Research conducted by Ping et al. demonstrated significantly lower levels of E-cadherin in ESCC tissues compared to normal tissues surrounding the cancer, confirming the impact of reduced E-cadherin on tumor metastasis (48). The mechanism underlying the decrease in E-cadherin expression appears to be closely related to microRNA regulation. Song et al. found that microRNA-9 promotes EMT progression by targeting and regulating E-cadherin through interaction with the 3’-untranslated region of E-cadherin (49). Liu et al. also discovered that microRNA-25 affects the expression of E-cadherin (50). Additionally, transforming growth factor β1 (TGFβ1) has been found to be negatively correlated with E-cadherin expression in tumors. TGFβ1 expression is higher in esophageal cancer tissues compared to normal squamous epithelium and nonmalignant Barrett’s mucosa. Rac3 protein ubiquitination and degradation can influence E-cadherin expression through the TGFβ1 pathway, providing further insights into the downregulation mechanism of E-cadherin (51) (**Figure 1**).

Integrins, also known as integrin, are Ca2+- or Mg2+-dependent heterophilic cell adhesion molecules that mediate mutual recognition and adhesion between cells and between cells and the extracellular matrix. They play a role in linking external cellular interactions to internal cellular structures. Integrins typically consist of two subunits, α and β, and approximately 20 different integrins have been identified (52). In the process of hematogenous metastasis, integrins play a significant role. On one hand, they regulate endothelial cell differentiation and migration (53) and promote tumor angiogenesis (54). On the other hand, in hematogenous metastasis, tumor cells with surface α2β1, α3β1, α6β1, and α6β4 integrins (55) promote tumor cell anchoring and penetration in the vascular endothelium by binding to laminin in the basement membrane. Platelets, mediated by αIIbβ3 integrin, aid in the aggregation and recruitment of plasma fibrin as well as tumor cells and fibrin within the vascular system. αvβ3 integrin binding to the fibrin-fibronectin complex on the surface of tumor cells rapidly activates, promoting tumor cell adhesion and penetration into the endothelium (56). Integrins associated with esophageal cancer metastasis include ITGβ1, ITGαV, ITGβ6, ITGα7, ITGα11, and others. Among them, ITGβ1 and ITGα7 also have important effects on lymphatic metastasis of esophageal cancer. One study found increased ITGβ1 expression in esophageal adenocarcinoma (EAC) with lymphatic metastasis (57), while ITGα7 may be involved in regulating stemness-related genes and reducing lymph node metastasis in esophageal squamous cell carcinoma (ESCC) through activation of the FAK/MAPK/ERK signaling pathway (58). Another study found that ITGαV accelerates MMP9 activation via the TGF-β pathway, promoting tumor cell migration and proliferation, although its relationship to EAC remains to be confirmed. In ESCC, Liu et al. discovered that ITGαV promotes tumor cell migration and proliferation through the downstream FAK/PI3K/AKT and TGFβ/SMAD2/3 signaling pathways, influencing tumor progression (59, 60). Additionally, Li et al. observed a positive correlation between ITGβ6 and HAX-1 expression levels in ESCC, suggesting that HAX-1 may regulate ESCC metastasis by modulating ITGβ6 activity (61). Ainiwaer et al. found that ALPK2, as a downstream target gene, promotes ESCC tumor cell migration in vascular endothelial cells by regulating ITGα11 (62) (**Figure 1**).

Selectins are a class of adhesion molecules that utilize glycosyl groups as recognition ligands. They were originally identified in leukocytes, platelets, and endothelial cells. The selectin family consists of three members: L-selectin, P-selectin, and E-selectin. The ligands of selectins are not only present on leukocytes but are also expressed on the surface of various types of tumor cells. Experimental evidence suggests that E-selectin mediates adhesion between tumor cells and endothelial cells during tumor metastasis. P-selectin triggers the activation of integrin αIIbβ3 by binding to the cytoplasmic structural domain of talin1, leading to platelet recruitment around circulating tumor cells (CTCs) and protecting them from destruction by natural killer (NK) cells (63). There is limited research on the relationship between selectins and esophageal cancer progression, which may be an important area for future investigation (**Figure 1**).

The TGFβ pathway typically involves the phosphorylation of type I receptors by TGFβ ligands upon binding to the TGF-βII receptor. This phosphorylation event leads to the phosphorylation of the C-terminus of Smad2/3 and the formation of the Smad2/3-Smad4 complex. The Smad2/3-Smad4 complex translocates to the nucleus, where Smad4 binds to Smad-related DNA sequences and regulates the transcription of TGF-β target genes (64). Besides the canonical Smad pathway, the TGFβ receptor complex can also activate the PI3K-AKT-TOR pathway and initiate the MAPK cascade (65). In early-stage esophageal squamous cell carcinoma (ESCC), TGFβ inhibits tumor progression by activating cell cycle suppressor genes. However, in advanced stages, the effect of TGFβ is reversed due to alterations in the Smad pathway of tumor cells. Tumor epithelial cells often exhibit Smad4 deficiency, leading to a compensatory increase in TGFβ during the late stages (66). Among the TGFβ isoforms, TGF-β1 has the greatest impact on ESCC progression (67) (**Figure 1**).

In the presence of these factors, tumor cells that have undergone epithelial-mesenchymal transition (EMT) can enter the bloodstream through the vascular endothelium (**Figure 1**).

##### 2.2.1.3 Ensuring the stability of the circulation and metastasis to distant organs

Tumor cells that enter the bloodstream are referred to as circulating tumor cells (CTCs). Typically, CTCs exist as individual cells, but in some cases, they can aggregate to form CTC clusters, which possess a more aggressive potential (68). However, CTCs in the circulation are susceptible to external influences such as immune cell attack, shear forces from blood flow, and anoikis, resulting in only a small fraction of CTCs successfully metastasizing to other organs (69). Recent evidence suggests that epithelial-mesenchymal transition (EMT) and its reverse process, mesenchymal-epithelial transition (MET), can induce phenotypic changes in CTCs in response to these external influences (70) (**Figure 1**).

Platelet adhesion and aggregation, along with the involvement of p-selectin, fibronectin (FN), and platelet granules (PG), contribute to the formation of CTC clusters. This process leads to the secretion of p-selectin and TGF-β1 through the TGF-β1-garp pathway. The adhesion and aggregation of platelets induce the synthesis of FN, providing mechanical protection against shear forces and immune cell attacks. Additionally, fibrin binding to leukocytes promotes the formation of small clots, enhancing the stability of CTC clusters and promoting their survival during hematogenous metastasis (71) (**Figure 1**).

Numerous studies have demonstrated that CTCs can serve as diagnostic indicators for esophageal cancer and provide insights into distant metastasis (72). Some studies have also suggested that CTCs can be predictive of the prognosis in patients with esophageal cancer. However, further research is still needed before CTCs can be effectively implemented as clinical indicators (73).

#### 2.2.2 Advances in tumor metabolism during lymphatic metastasis

The process of lymphatic metastasis shares similarities with bloodstream metastasis. It involves several steps, starting with the formation of a premetastatic niche in the lymph nodes and the growth of lymphatic vessels to prepare for metastasis. The second step involves adhesion to the extracellular matrix, invasion of lymphatic vessels near the primary site, and migration to the draining lymph nodes. The third step is evading the body’s immune response and maintaining the stability of the lymph nodes. Finally, systemic metastasis occurs through the lymphatic vessels.

In the first step, the components of the premetastatic niche in lymph node metastasis include extracellular vesicles (EVs) responsible for substance transport, tumor-derived lymph nodes and lymphatic vessels, fibroblasts, and various growth factors secreted by them.

One important growth factor involved in the tumor microenvironment and lymphatic vessel development is VEGF. In addition to VEGF-A and VEGF-B, VEGF-C and VEGF-D play a major role in promoting lymphangiogenesis. They bind to VEGFR-3 and activate the ERK1/2 and PI3K-AKT pathways, which can affect lymphangiogenesis and lymphatic endothelial cell proliferation (74). VEGF-C, in particular, has been implicated in promoting distant metastasis by inducing lymph node formation. It may also contribute to lymphatic metastasis by degrading calmodulin in lymphatic endothelial cells (75). The expression levels of VEGF-C and VEGF-D in esophageal cancer are closely associated with the TNM stage of the disease (76). Current research on lymphatic metastasis and VEGF has focused on identifying substances that target and regulate VEGF-C levels, which could provide a basis for future targeted therapies for esophageal cancer. For instance, nicotine has been shown to increase VEGF-C levels in ESCC and influence lymphatic metastasis by reducing the expression of OTUD3, thereby inhibiting the ability of ZEP36 to bind to specific regions of VEGF-C mRNA and causing its decline (77). Endostatin has also been found to decrease VEGF-C production by affecting VEGF-C mRNA (78). Additionally, OCT4 has been shown to increase VEGF-C production and induce the EMT process in ESCC by activating the VEGF-C/VEGFR-3 pathway.

In addition to VEGF, TGF-β and FGF2 also play a role in lymph node metastasis. TGF-β mainly induces EMT, which affects lymphatic metastasis, and the mechanism has been mentioned in the context of bloodstream metastasis. FGF-2 promotes ESCC cell metastasis and stimulates endothelial cell production by binding to the FGFR-1 receptor and activating the PI3K-AKT and ERK1/2 signaling pathways (79).

Exosomes produced by tumors, including their cargo of miRNAs and lncRNAs, have been shown to impact the immune response and contribute to immune escape, which is crucial for lymphatic metastasis (80). MiRNAs and lncRNAs derived from exosomes can influence the process of lymphatic metastasis in esophageal cancer. For instance, exosome-mediated miR-203 was found to be significantly upregulated in ESCC with lymphatic metastasis compared to the group without lymphatic metastasis (81), indicating its association with lymphatic metastasis. The mechanism may involve promoter methylation of miR-203, which affects its expression and leads to uncontrolled expression of downstream target gene LASP1, thereby influencing lymphatic metastasis in ESCC. However, this mechanism has not been confirmed (82). Furthermore, miR-21 overexpression was shown to significantly inhibit the invasive and metastatic abilities of esophageal cancer and reduce its sensitivity to chemotherapy (Influence of exosome-derived miR-21 on chemotherapy resistance of esophageal cancer). A recent study in 2023 discovered that exosome-derived microRNA-10527-5p is involved in the process of esophageal cancer lymph node metastasis by blocking the Rab10-mediated Wnt signaling pathway, inhibiting the EMT process, and controlling VEGF-C production to suppress lymphangiogenesis (83). Additionally, exosome-derived lncRNAs can induce apoptosis, promote immune escape of tumors in lymphatic tissues, and affect antigen presentation processes, as well as contribute to angiogenesis, as mentioned earlier (84). For example, recent studies have found that lncRNA LINCO2820 selectively splices pre-RNA-related genes, amplifies the NF-kB signaling pathway, and promotes ESCC metastasis by interacting with splicing-related regulator SF3B3 under TNFα stimulation. This suggests that lncRNA LINCO2820 could be a potential target for the treatment of metastatic ESCC (85).

##### 2.2.2.1 Lymphatic vessels and lymph nodes

Lymphatic vessels primarily consist of lymphatic endothelial cells, and there are three main sources of endothelial cells in tumor lymphatic vessels: first, they can arise from further proliferation of the original lymphatic vessels; second, they can result from the transformation of vascular endothelial cells; and third, they can arise from the transdifferentiation of other cell types. Some known cells involved in this process are mesenchymal stem cells (86) and tumor-associated macrophages (87). Neoplastic lymphatic vessels and lymph nodes play a crucial role in tumor metastasis. On one hand, lymphatic vessels and lymph nodes can serve as sites for distant metastasis, and on the other hand, tumor-induced lymphatic vessels and lymph nodes create a favorable microenvironment for metastatic cells by facilitating the transport and storage of tumor antigens and effector cells, thereby promoting their growth (74). Lymphatic endothelial cells in neoplastic lymph nodes and lymphatic vessels contribute to immune evasion of tumors by presenting tumor antigens and inducing apoptosis of CD8+ cells (88). Additionally, lymphatic vessels can induce the aggregation of cancer cells and immune cells through the release of chemokines.

##### 2.2.2.2 CAFs

With the secretion of relevant growth factors, tumor cells promote the aggregation and conversion of adjacent fibroblasts into tumor-associated fibroblasts (CAFs) (89). CAFs play a significant role in the establishment of the premetastatic microenvironment and contribute to lymphatic metastasis. On one hand, CAFs can stimulate lymphatic vessel formation and promote the process of epithelial-mesenchymal transition (EMT) by secreting growth factors such as VEGF, FGF, and TGF-β, thus facilitating lymphatic metastasis. On the other hand, CAFs can disrupt the extracellular matrix (ECM) by releasing protein hydrolases, ensuring the release of the aforementioned growth factors and promoting cancer cell invasion and dissemination (90). For example, a study published in 2021 revealed that CAFs are connected to lymphatic-specific carriers (LSCs) through paracrine interactions involving fibrinogen activator inhibitor (PAI-1) and the LRP1 protein, triggering the AKT and ERK pathways and promoting metastasis in esophageal squamous cell carcinoma (ESCC) cells and macrophages (91). Furthermore, CAFs can counteract the immune response by promoting immune cell aggregation. Specifically, CAFs can secrete chemokines that attract immunosuppressive cells into the tumor microenvironment, thereby modulating the tumor phenotype and facilitating the acquisition of immunosuppressive abilities by tumor cells. For instance, CAFs can promote neutrophil aggregation by releasing CXCL-like chemokines and induce monocyte aggregation and polarization through the release of MCP-1 (92). Moreover, CAFs have been found to inhibit the activation of immune cytotoxic cells or modulate their function, thereby preventing these cells from eliminating tumor cells. For example, CAFs can release TGF-β, which reduces the expression of NK cell receptors and inhibits the cytotoxic effect of NK cells (93, 94). Limited research has been conducted on CAFs in the context of esophageal cancer. Huang et al. found that in ESCC patients, the amount of WNT2+ released by CAFs was inversely correlated with CD8+ T-cell activity. They demonstrated that WNT2+ inhibits the activation of the JAK2/STAT3 signaling cascade by affecting the secretion of SOCS3, thereby suppressing T-cell differentiation and expression and promoting immune evasion in ESCC. Liao et al. found an association between reduced levels of CAF-released FAPα and ESCC (95). A study from Japan also revealed that CAFs can promote the growth and migration of macrophages and ESCC cells, as well as induce the polarization of M2 macrophages, by secreting cytokines such as CCL2, CXCL8, and IL-6. Moreover, CAFs release FAP, which activates AKT- and ERK-related signaling pathways, leading to the production of the aforementioned cytokines and the development of immunosuppressive properties in ESCC cells (96).

**Figure 2:**
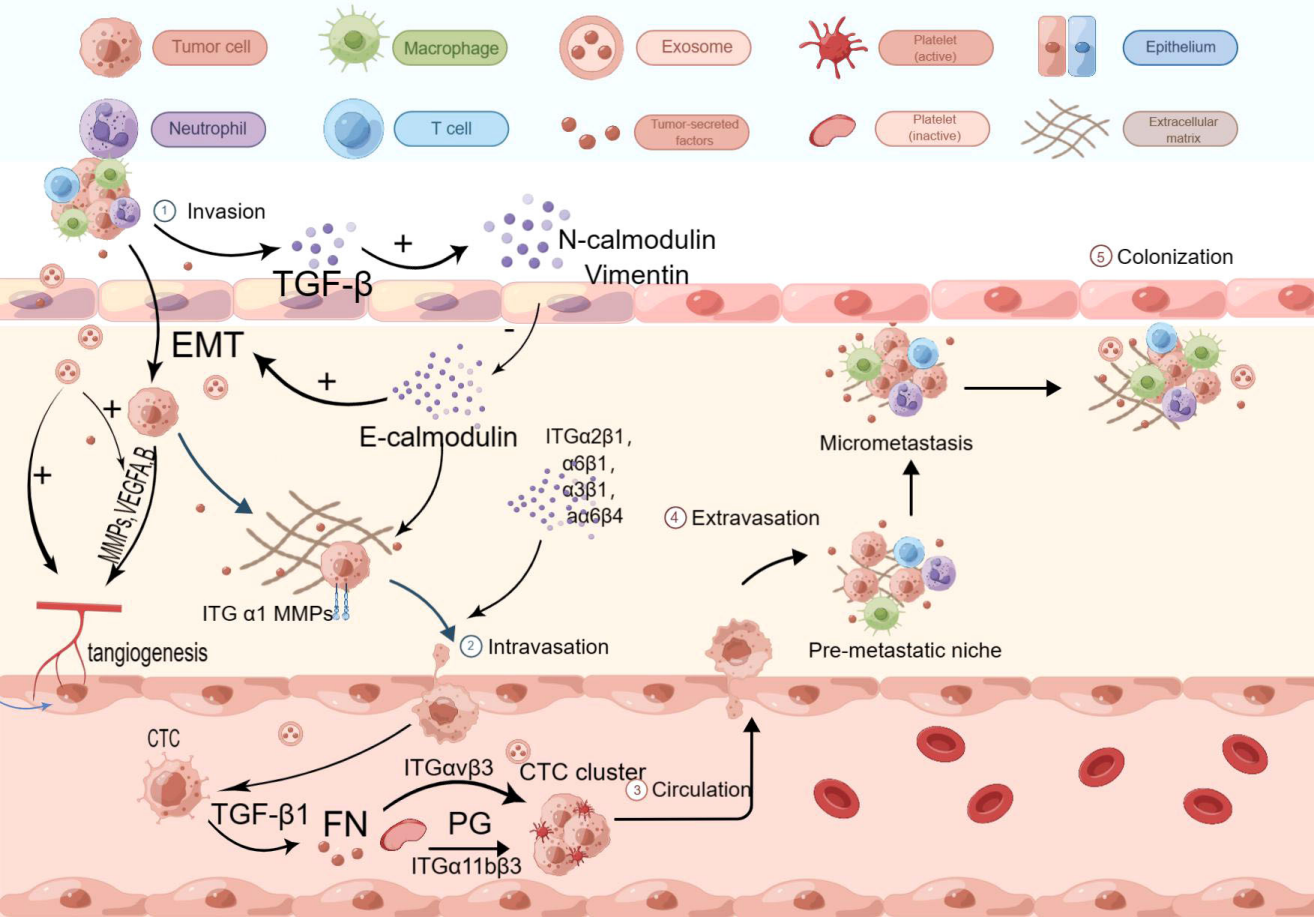
Immunosuppressive mechanisms of EC. Tumor cells release TGF-β, activating CAFs, Tregs, and TAMs. CAFs release CXCL-like chemokines, promoting neutrophil aggregation. CAFs also release MCP-1, promoting monocyte aggregation and transformation. Furthermore, CAFs release FAP, activating AKT- and ERK-related signaling pathways, and promoting the release of CCL2, CXCL8, and IL-6. The amount of WNT2+ released from CAFs is inversely correlated with CD8+ T cell activity. WNT2+ inhibits the JAK2/STAT3 signaling cascade by affecting SOCS3 secretion, suppressing T cell differentiation and expression. CCL2, CXCL8, IL-4, IL-6, and IL-13 induce M2 macrophage polarization and TAM aggregation. M2 macrophages deplete CD8+ T cells, which have specific antitumor effects, and enhance the PD-1/PD-L1 pathway, increasing tumor cell immune escape. CCL17, CCL20, CCL21, and CCL22 promote Treg aggregation. Treg cells express high-affinity IL-2 receptors on their surface, competitively binding and depleting IL-2, inhibiting effector T cell proliferation. Treg cells secrete suppressive cytokines, including IL-32 and TGF-β, to inhibit T cell activation. Treg cells also secrete CTLA-4, reducing CD80/86 expression on APCs and inhibiting APC maturation, impairing immune function within the TME.

In the second step of lymphatic metastasis, cancer cells traverse lymphatic endothelial cells in a manner similar to bloodstream metastasis, both of which involve the process of EMT. Unlike hematogenous metastasis, lymphatic metastasis involves the release of chemokines from endothelial cells and the binding of related receptors to facilitate the translocation of tumor cells to lymph nodes (97). For example, lymphatic endothelial cells (LECs) can release CCL21, which attracts ESCC cells and immune cells via the CCL21-CCR7 axis. It has been suggested that the CCL21-CCR7 pathway stimulates increased expression of MUC1 through the ERK1/2-Sp1 pathway, promotes the release of MMP13, and contributes to lymphatic metastasis development (98). The CXCL12-CXCR4 pathway is also crucial in the chemotactic function of lymphatic metastasis in esophageal cancer. Studies have found that the CXCL12-CXCR4 pathway may promote MMP-9 secretion and stimulate lymphatic metastasis through the phosphorylation of the ERK1/2 pathway (99). Moreover, apart from its role in mediating metastasis, the CXCL12-CXCR4 pathway also facilitates the trafficking of T cells and dendritic cells, thereby aiding immune escape from tumors (100). Furthermore, it has been demonstrated that the CXCL12-CXCR4 pathway, through activation of the STAT3 pathway involved in EMT, can accelerate the progression of lymphatic metastasis (101). Additionally, the CCL2-CCR2 pathway has been identified as capable of inducing the aggregation of monocytes and macrophages, leading to the conversion of tumor-associated macrophages (TAMs) into M2-type macrophages, which promote cancer progression and impact lymph node metastasis (102) (**Figure 2**).

#### 2.2.3 Mechanisms of immune escape

In the final step, ensuring immune evasion of tumors is crucial for the success of metastasis. Within the tumor microenvironment (TME), CD8+ T cells play a crucial role in antitumor immunity. However, M2 macrophages, cancer-associated fibroblasts (CAFs), and regulatory T cells form an immune barrier that hampers the CD8+ T cell-mediated antitumor immune response and prevents effective killing of tumor cells (103).

Macrophages within the TME can differentiate into M1 or M2 phenotypes, with the influence of CAFs and Th2 cells leading to the release of cytokines that promote M2 macrophage polarization. Notably, IL-4 and IL-13 have been identified as promoters of M2 macrophage polarization (96). Programmed death receptor-1 (PD-1) has been found to play a crucial role in the immune evasion of esophageal cancer cells regulated by tumor-associated macrophages (TAMs). The PD-L1 pathway-mediated antitumor immune response has been a key focus of current research. Polarization of TAMs towards the M2 phenotype within the tumor microenvironment can reduce the number of CD8+ T cells involved in specific antitumor effects by engaging the PD-1/PD-L1 pathway, thereby increasing the likelihood of tumor immune evasion. Furthermore, it has been observed that M2 macrophages upregulate PD-L2 receptor expression, further enhancing the immunosuppressive environment in ESCC (104) (**Figure 2**).

The effect of CAFs on immunosuppression has been described in detail above and therefore will not be repeated.

Regulatory T cells (Tregs) are a subset of CD4+ T cells (105) that play a role in suppressing excessive immune responses within the body. In the tumor microenvironment, both intrinsic and induced Treg cells can undergo phenotypic and functional changes and interconversion (106). Treg cells are important components involved in suppressing tumor immune responses in cancer patients. They directly inhibit the function of CD4+ T cells, CD8+ T cells, macrophages, NK cells, and DC cells. Tregs express a high-affinity IL-2 receptor on their surface, which competitively binds and consumes IL-2, leading to the death of CD4+ T cells and inhibiting the activation of CD8+ T cells (107). Tregs inhibit T cell activation by secreting inhibitory cytokines such as IL-10 and TGF-β, or by inducing other cells to secrete these cytokines. One of the main mechanisms involves cTLA-4, which has been implicated in the immunosuppressive function of Tregs. Studies have shown that CTLA-4 expressed by Tregs can stimulate CTLA-4-dependent macrophages to remove CD80/CD86 molecules on antigen-presenting cells (APCs). Reduction of CD80/CD86 inhibits the activation of naïve T cells and disrupts the CD80/PD-L1 heterodimer, increasing free PD-L1 and inhibiting the activation of effector T cells expressing PD-L1 (108). Furthermore, CTLA-4 on Tregs has a higher affinity for CD80/CD86 on APCs compared to CD28 molecules on T cells. This competitive binding of CTLA-4 to CD80/CD86 inhibits CD28 stimulation on T cells and modulates APC activation (108–110) (**Figure 2**).

The role of Treg cells in esophageal cancer is an active area of research. The transcription factor Eomesodermin has been found to promote the production of CCL20, which binds to CCR6 and enhances Treg aggregation in the tumor microenvironment, thereby accelerating the proliferation of ESCC. Additionally, CCL17 and CCL21 can bind to CCR4 to recruit Tregs (109, 111). IL-32 has been discovered to activate CD8+ T cells, induce IFN production, and slow down ESCC progression, while simultaneously increasing the immunosuppressive activity of Tregs by promoting Foxp3 expression (112). IL-33 has been found to promote the NF-κB signaling pathway through CCL22 expression, attracting Tregs into the tumor microenvironment of ESCC and affecting its progression (113). Furthermore, Fusobacterium nucleatum has been implicated in promoting ESCC progression by inducing Treg aggregation, although the precise mechanism is not yet fully understood (114).

Myeloid-derived suppressor cells (MDSCs) are a highly heterogeneous class of myeloid-derived cells with mononuclear and polymorphic nuclei. They are derived from myeloid progenitor cells (CMP) located in the bone marrow (or mouse spleen). In the context of tumor pathology, the differentiation of immature myeloid cells is blocked, leading to the development of MDSCs (115). MDSCs primarily contribute to immunosuppression by inhibiting the immune response of T cells and NK cells and promoting Treg activation. MDSCs can be divided into two main subsets: granulocytic/polymorphonuclear MDSCs (PMN-MDSCs) and monocytic MDSCs (M-MDSCs). The STAT3 pathway can activate downstream targets and stimulate PMN-MDSCs to release arginase-1, which depletes arginine involved in T cell immune response, thereby inhibiting the expression of the CD3ζ chain and affecting T cell activation (116). M-MDSCs inhibit T cell function by binding and regulating nitric oxide synthase-2, which inhibits JAK3/STAT5 signaling and MHCII expression. Studies have found that IL-6 and CXCL16 can induce the expression of CD38 on the surface of MDSCs. MDSCs with high expression of CD38 exhibit enhanced binding ability to nitric oxide synthase-2 and stronger inhibitory effects on T cell activation (117). Additionally, Th2 cytokine IL-4 has been found to impact the distribution of MDSCs and enhance the immunosuppressive ability of MDSCs by increasing arginase-1 activity (118). Apart from these pathways, MDSCs can inhibit T cell immune function by increasing reactive oxygen species (ROS), peroxynitrite, prostaglandin E2 (PGE2), and suppressing the expression of immune regulatory molecules such as PD-L1 (119).

In addition to these immunosuppressive cells, the cytokines they secrete, including TGF-β, interleukins, and chemokines, also play important roles. TGF-β is a key activator and transformer of immune cells and significantly contributes to immunosuppression. As mentioned earlier, TGF-β indirectly promotes immunosuppression by activating Tregs, stimulating the transformation of peripheral fibroblasts into cancer-associated fibroblasts (CAFs), and inducing the differentiation of TAMs into M2-type macrophages. Interleukins (ILs) are crucial regulators involved in mediating inflammatory responses and regulating immune cells (120). ILs associated with immunosuppression in esophageal cancer include IL-4, IL-6, IL-10, IL-32, and IL-33. The roles of IL-32 and IL-33 have been described previously. In the tumor microenvironment, IL-4 primarily suppresses the activation of Th1 cells and cytokine secretion. It has also been reported that IL-4 plays an important regulatory role in the polarization of TAMs toward M2 macrophages (121). A study found that the rs2243263 G>C SNP of the IL-4 gene is closely related to the development of ESCC. IL-6 signals by binding to the IL-6R/CD126 receptor complex on the cell surface, which, in conjunction with the signal transduction component gp130 (CD130), activates the JAK2/STAT3 pathway. STAT3 promotes PD-L1 expression, thus contributing to tumor immunosuppression. Metformin has been found to inhibit this mechanism, hindering ESCC proliferation (122). IL-6 can also promote the release of CXCL12, inducing the aggregation of immunosuppressive cells and accelerating the epithelial-mesenchymal transition (EMT). ING5 has been shown to influence ESCC progression by inhibiting this process (123). Moreover, IL-6 can stimulate the release of more ROS and iNOS from myeloid-derived suppressor cells (MDSCs), inhibiting NK cell activation and promoting TAM formation (124). IL-10 is generally released by TAMs in the tumor immune microenvironment and contributes to immunosuppression by affecting Treg subpopulations and promoting the release of immunosuppressive factors, including TGF-β (125). Other IL molecules can also suppress T cell responses and facilitate immune evasion by tumor cells (126). IL-10 can stimulate the release of HLA-G, which, in turn, promotes the production of more IL-10 by Th2 helper cells, creating positive feedback. HLA-G can assist tumor cells in evading immune cell surveillance by promoting the release of MMPs. One study found that IL-10 stimulated HLA-G production and induced the release of MMP-21, impacting the immunosuppressive process in ESCC (127). Another study discovered that IL-10 and PD-L1 levels were higher in ESCC compared to normal tissues, and these two variables were positively correlated. The authors suggested that IL-10 may regulate PD-L1 expression through negative feedback via the MET signaling pathway, inhibiting cellular immune effects (128).

### 2.3 Special mechanism of esophageal cancer metastasis

Ferroptosis mechanisms have emerged as a significant area of research in cancer studies in recent years. Ferroptosis is a specific type of programmed cell death characterized by the accumulation of lipid peroxides due to increased intracellular iron levels. The breakdown of lipid peroxides generates reactive oxygen species that can damage intracellular macromolecules and accelerate cell death. A 2023 study proposed a link between ferroptosis mechanisms and lymph node metastasis in ESCC, suggesting that ESCC cells employ certain mechanisms to inhibit or evade the process of ferroptosis, thereby promoting lymphatic metastasis. Their research revealed that the key molecular mechanism crucial for lymphatic metastasis in ferroptosis is the overexpression of BACH1. BACH1, on one hand, suppresses the transcription of endoplasmic reticulum catalase SCD1, thus inhibiting the synthesis of oleic acid in tumor cell membranes. This creates a concentration gradient between tumor cells and lymphatic fluid, which contains high levels of oleic acid, thereby facilitating the metastasis of tumor cells to lymphatic vessels. On the other hand, BACH1 hinders the accumulation of lipid peroxides, thereby reducing the occurrence of ferroptosis (129). Interestingly, in other studies, oleic acid has been found to activate ACSL1, an enzyme that binds to membrane phospholipids and inhibits the ferroptosis process (130). Additionally, variations in microRNAs have been found to influence the progression of ferroptosis. A study conducted in 2022 revealed that hypoxia induces the production of E2F7 and activates the splicing factor QKI, which leads to increased production of microBCAR3 and subsequent upregulation of the transport protein TNP1. This process acts to inhibit ferroptosis (reference).

### 2.4 The role of immune cells in tumor metastasis

Many studies have highlighted the significant role of immune cells in the tumor microenvironment in tumor metastasis. For instance, tumor cells secrete TGF-β, which promotes the polarization of neutrophils to the N2 subtype. N2 neutrophils can release MMP-9 and VEGF to participate in angiogenesis and also secrete IL-10, CCL2, and other molecules involved in mediating immunosuppression (131). A recent study found that the neutrophil-associated serine protease Cathepsin G can enhance the cell adhesion of E-cadherin by activating insulin-like growth factor 1, facilitating tumor cell aggregation, and aiding tumor cells in penetrating blood vessels (132). Interestingly, neutrophils can also inhibit tumor metastasis. Studies have found that increased expression of TRPM2 during epithelial-mesenchymal transition (EMT) makes mesenchymal cells more susceptible to neutrophil-mediated cytotoxicity, thereby inhibiting the EMT process (133).

Macrophages can promote cell migration by interacting with tumor cells. Previous research has demonstrated that tumor cells synthesize CSF-1 to induce macrophage migration, and these macrophages can secrete EGF to stimulate tumor cell migration. Other chemokines such as CXCL12 can also initiate this co-migration process, but CSF-1 and EGF are still required for subsequent signal transduction (134). Additionally, macrophages enhance the invasive capacity of tumor cells. For example, M2 macrophage-derived exosomes have been found to contain a high concentration of ITGαVβ3, which activates the FAK signaling pathway between cells and enhances the invasive ability of tumor cells (135). Furthermore, macrophages in different locations exert diverse effects on tumor metastasis. Omental macrophages, for instance, can secrete CCR1-related ligands that promote the metastasis of ovarian and digestive tract malignant tumors to the cell clusters on the surface of the greater omentum, facilitating tumor cell colonization (136). Peritoneal macrophages are involved in oxidative phosphorylation and fatty acid oxidation during peritoneal metastasis. The metabolite itaconic acid produced in this process can promote peritoneal metastasis (137). Simultaneously, the secretion of VEGF by peritoneal macrophages can increase the permeability of lymphatic vessels and blood vessels, leading to the production of ascites during metastasis. Ascites and VEGF create an optimal growth environment for tumor cells (138). In addition to promoting tumor cell migration and angiogenesis, M2 macrophages can induce the secretion and expression of cytokines such as IL-10 and M-CSF by releasing FGL2, thereby promoting the polarization of macrophages toward the M2 phenotype. Moreover, FGL2 can induce the expression of CD39 and facilitate the transition of M1 macrophages to M2 macrophages (139). It is known that M2 macrophages secrete a wide range of cytokines, participate in angiogenesis, immunosuppressive processes, and more. For example, M2 macrophages can transfer ITGαMβ2 to liver cancer cells via exosomes, activate MMP-9 expression, and promote angiogenesis (140). M2 macrophages can also induce an inflammatory response and facilitate tumor growth by secreting inflammatory factors such as TGF-β, IL-10, and IL-6 (141).

## 3 Advances in the treatment of esophageal cancer metastases

For patients with advanced metastases, the risks associated with surgical treatment are higher, and the prognosis is generally poor. Consequently, surgery is no longer considered the primary treatment option for metastatic esophageal cancer. Instead, a multidisciplinary approach involving chemotherapy, radiotherapy, immunotherapy, and targeted therapy should be considered.

### 3.1 Chemotherapy

Esophageal cancer exhibits relatively high sensitivity to chemotherapy, but the use of chemotherapy alone is no longer sufficient to achieve favorable outcomes in cases of metastatic esophageal cancer. Therefore, the key to improving the survival rate of patients with advanced disease lies in the design of rational chemotherapy regimens and courses, as well as the effective integration of other treatment modalities (142). Neoadjuvant chemotherapy, a standardized approach for middle and advanced esophageal cancer, aims to reduce the tumor size, lower the pathological stage, and eliminate early metastases. Patients who respond well to neoadjuvant chemotherapy may undergo surgery, which can contribute to improved 5-year survival rates (143).

#### 3.1.1 Chemotherapy regimen

Chemotherapy regimens for advanced esophageal cancer have seen limited changes in recent decades. Commonly used drugs include 5-fluorouracil (5-FU) and cisplatin, often combined with capecitabine, S-1, or paclitaxel-like drugs such as paclitaxel and docetaxel. Research in chemotherapy regimens has shifted towards exploring multidrug combinations and combinations with other therapies to enhance treatment effectiveness (144, 145).

Among various chemotherapy regimens, the combination of docetaxel, cisplatin, and 5-fluorouracil (DCF) is considered highly effective. Studies have shown that presurgical DCF regimens exhibit significant antitumor activity, improve the success rate of local metastatic surgery for stage II/III esophageal cancer, and prolong patient survival. A recent Japanese study investigated the DCF regimen in 48 patients with stage T4 or supraclavicular lymph node metastases. The study found higher 1-year survival rates compared to the CF-RT regimen for advanced tumors and a significant reduction in postoperative complications. However, the long-term survival benefits of the DCF regimen for metastatic patients remain unclear as the study was not followed up to 5 years (146).

Furthermore, Zheng et al. conducted a study comparing the efficacy of cisplatin and lopressor (LBP) in esophageal cancer. They evaluated 733 patients with intermediate to advanced esophageal squamous cell carcinoma (ESCC) and compared the 5-year survival rate and complications between the cisplatin and LBP groups. The results showed similar 5-year survival rates between the LBP and cisplatin groups, but the incidence of post-chemotherapy side effects was lower in the LBP group. This suggests that LBP combined with docetaxel may be another effective neoadjuvant chemotherapy regimen for ESCC. Additionally, studies have suggested the effectiveness of the docetaxel + nedaplatin combined with S1 (DGS) regimen for the treatment of intermediate to advanced esophageal cancer (147).

The efficacy of paclitaxel in combination with 5-FU and cisplatin for advanced esophageal cancer has been shown to be significantly improved and comparable to that of docetaxel. Neutropenia was found to be more common in the docetaxel group, which may guide the selection of regimens for specific patients. A Japanese study also evaluated the efficacy difference between paclitaxel and irinotecan and found similar effects, suggesting that both could be used as second-line agents in chemotherapy (148, 149).

#### 3.1.2 Effectiveness of chemotherapy

In recent years, there has been growing interest in studying the factors that influence the effectiveness of chemotherapy. For instance, Zeng et al. discovered that HNL1 could promote the expression of tumor-associated proteins in ESCC by enhancing PLK1 transcription, thereby reducing the sensitivity to chemotherapeutic drugs. Consequently, combining chemotherapy with BL-2356, a targeted medication that inhibits PLK1 transcription, could significantly enhance its effectiveness (150). Lu et al. found that aberrant expression of the Yap1 gene could confer resistance to tumor chemotherapy by affecting EGFR expression (151). Furthermore, specific infections in combination with ESCC can also impair the efficacy of chemotherapy. For instance, Gao et al. found that P. gingivalis infection renders esophageal cancer cells resistant to chemotherapy-induced apoptosis, thereby reducing the effectiveness of chemotherapy (152). However, certain conservative measures might improve the prognosis of chemotherapy. Li et al. compared the incidence of chemotherapy complications between patients who received adequate oral nutritional supplementation (ONS) and control patients and found that ONS may reduce the occurrence of chemotherapy-induced myelosuppression, particularly in patients with a BMI ≤18.5 kg/m2 (153).

In addition to the influencing factors, studies on prognostic assessment indicators of chemotherapy can inform future treatment strategies. A Japanese study evaluated the prognostic factors influencing recurrence-free survival (RFS) and overall survival (OS) after neoadjuvant chemotherapy combined with surgery. The results revealed that preoperative SCC-A values, TNM staging of residual tumors after neoadjuvant chemotherapy, lymphovascular and vascular invasion, and supraclavicular lymph node metastasis significantly impacted the prognosis of neoadjuvant chemotherapy combined with surgery. Patients with a poor prognosis may require additional postoperative adjuvant therapy (154). Similar findings were reported in another study, which demonstrated a correlation between lymphatic invasion, post-chemotherapy residual lymph node metastases, and the prognosis of neoadjuvant chemotherapy followed by surgery (155).

### 3.2 Immunotherapy

As an emerging treatment, immunotherapy can be classified into four categories: immune checkpoint inhibitors (such as PD-1/L1 inhibitors), tumor vaccines (e.g., provenge, cimavax), cellular immune cell therapy (CAR-T), and non-specific immunomodulators. Several studies have shown that immunotherapy alone has a superior antitumor effect compared to chemotherapy in advanced and metastatic esophageal cancer. Furthermore, combining immunotherapy with chemotherapy has been found to be more effective than immunotherapy alone. However, it is important to note that the combination of these two treatments may also impose a greater burden on the patient’s body (156, 157).

#### 3.2.1 Immune checkpoint inhibitors

Immune checkpoint inhibitors work by reducing the immunosuppressive capability of tumors and stimulating immune T cells to target and eliminate tumor cells through inhibiting the interaction between PD-L1 on tumor cells and PD-1 on immune cells (158). PD-1 inhibitors are the most well-known among them. When combined with chemotherapy, PD-1 inhibitors can enhance the immune response and improve the ability to combat tumor immune evasion (159). Some of the latest PD-1 inhibitors include nivolumab, camrelizumab, sintilimab, tislelizumab, and pembrolizumab. In a 2018 study comparing nivolumab to second-line chemotherapy drugs such as paclitaxel, nivolumab demonstrated superior safety and effectiveness, with lower median overall survival and incidence of adverse events compared to chemotherapy (160). Another study in 2020 evaluated the efficacy of camrelizumab + apatinib in combination with paclitaxel + nedaplatin in advanced ESCC and found significantly higher median progression-free survival (PFS) and duration of response (DoR) compared to paclitaxel combined with nedaplatin alone, indicating better efficacy. However, this regimen may lead to increased side effects such as myelosuppression and liver damage, necessitating further research (161). The ORIENT-15 study divided 659 patients into control and sintilimab groups, both receiving paclitaxel + cisplatin chemotherapy. The experimental group showed significantly improved overall survival (OS) and PFS compared to the control group, suggesting the efficacy and safety of sintilimab in combination with paclitaxel + cisplatin (162). In 2022, He et al. investigated the efficacy and safety of tislelizumab in combination with neoadjuvant chemotherapy in patients with relapse after neoadjuvant radiotherapy for advanced ESCC. This regimen effectively reduced the recurrence and metastasis rates after surgery in the study participants (163). Comparing the pembrolizumab group to the chemotherapy groups receiving paclitaxel, doxorubicin, and irinotecan, a 2021 study by Cao et al. revealed that the pembrolizumab group had a lower incidence of adverse events and superior efficacy (164). PD-L1 inhibitors are also crucial in immunotherapy. In a 2021 study, the combination of the anti-PD-L1 antibody SHR-1316 with irinotecan + 5-Fu showed improved progression-free survival (PFS) compared to first-line chemotherapy regimens, including FP regimens, and had a lower incidence of adverse events. These findings indicate the efficacy and safety of this regimen, but further challenges need to be addressed before its clinical application (165). In 2023, Li et al. investigated the efficacy of the PD-L1 inhibitor Socazolimab in combination with paclitaxel plus cisplatin in advanced ESCC. They compared the major pathological remission rate (MPR) and pathological complete remission rate (pCR) between the Socazolimab and placebo groups after surgery. The Socazolimab group showed significantly higher MPR and pCR rates, as well as a decrease in T stage, suggesting that the combination of PD-L1 inhibitor Socazolimab with paclitaxel plus cisplatin can improve the success rate of R0 resection and enhance the survival rate of patients with advanced esophageal cancer (166).

#### 3.2.2 Influencing factors of immunotherapy

Many studies have consistently demonstrated the impact of PD-L1 expression on the efficacy of PD-1 inhibitors and PD-L1 inhibitors. The KEYNOTE-590 study revealed that pembrolizumab showed greater efficacy in patients with a PD-L1 combined positive score (CPS) ≥10 compared to those with CPS <10, leading to improved overall survival (OS) and progression-free survival (PFS) compared to chemotherapy alone in patients with PD-L1 CPS ≥10 (167). Similarly, a study in 2023 reported that the anti-PD-1 antibody serplulimab exhibited enhanced effectiveness in patients with high PD-L1 expression (168). Consistent with these findings, the ORIENT-15 trial, mentioned earlier, also indicated improved efficacy of sintilimab in patients with esophageal squamous cell carcinoma (ESCC) and PD-L1 CPS ≥10 (162).

It is worth noting that chemotherapy regimens can influence PD-L1 expression. For instance, one study suggested that cisplatin and 5-fluorouracil may enhance the immunotherapeutic effect by upregulating PD-L1 expression, possibly through modulation of the JAK/STAT pathway (169). However, further research is needed to elucidate the exact underlying mechanism. In addition to PD-L1, PD-L2 expression may also impact immunosuppression. Therefore, using PD-L1 and PD-L2 expression as markers to predict immunotherapy response has become a clinical trend (170). The process of ferroptosis may also have implications for immunotherapy effectiveness, although the specific mechanism remains unknown.

### 3.3 Targeted therapy

Targeted therapy involves inducing tumor cell-specific death using monoclonal antibodies that recognize tumor-specific antigens or epitopes. It is characterized by its ability to precisely target tumor cells while sparing surrounding normal tissue cells. In recent years, targeted therapy has emerged as a prominent area of research for treating advanced and metastatic esophageal cancer.

#### 3.3.1 Monoclonal antibodies

The monoclonal antibodies currently considered applicable in esophageal cancer include monoclonal antibodies that inhibit the epidermal growth factor receptor (EGFR) (panitumumab, cetuximab), monoclonal antibodies that inhibit the HER2 receptor (trastuzumab, pertuzumab), anti-PD-L1 antibodies, and monoclonal antibodies that inhibit the action of VEGF (ramucirumab). Panitumumab is mainly used for the treatment of colorectal cancer, and its mechanism mainly involves binding to EGFR receptors, preventing them from binding to EGF or TGF-α, thus inhibiting the EMT and proliferation process of cancer cells (171). A 2020 study suggested that an anti-EGFR monoclonal panitumumab-targeted regimen combined with oxaliplatin + epirubicin chemotherapy was less effective than expected in patients with EGFR-amplified esophageal cancer, and the investigators suggested that the poor results of this regimen may be due to a possible antagonistic relationship between anthracyclines, especially epirubicin, and panitumumab (172).

The SAKK 75/08 trial, published in 2018, randomized 300 patients to radiotherapy and surgery with or without cetuximab and analyzed PFS and OS values in both groups. The results showed a significant improvement in OS values in the cetuximab group but no significant difference in PFS values, as well as a reduction in the local recurrence rate after surgery. This result suggests a role for cetuximab in combination with radiotherapy and surgery in locally advanced resectable esophageal cancer, but the effect was not significant (173). Additionally, the difference in combination chemotherapy regimens affects the effect of cetuximab, which is similar to panitumumab mentioned above. The CALGB 80403 trial compared changes in OS and PFS after three different chemotherapy regimens combined with cetuximab, and the findings suggest that epirubicin, cisplatin, and continuous infusion fluorouracil (ECF), irinotecan + cisplatin (IC), or FOLFOX (oxaliplatin, calcium folinate, injection and infusion fluorouracil) among IC in combination with cetuximab were the least effective with a high likelihood of subsequent adverse events, while the ECF and FOLFOX regimens had similar efficacy, but FOLFOX was less likely to require subsequent treatment (174). Interestingly, a study from 2020 suggested that the regimen of cetuximab combined with oxaliplatin plus 5-Fu had strong side effects with deaths attributed to ARDs, contradicting the above findings, and the investigators suggest that adding a radiotherapy course to the regimen may reduce the cetuximab and anthracycline effects of pulmonary toxicity caused by the combination of cetuximab with anthracyclines. In conclusion, much of the literature suggests a role for anti-EGFR receptor monoclonal antibodies in the treatment of advanced esophageal cancer, but the extent of the effect depends on the combination chemotherapy regimen.

Anti-HER2 receptor monoclonal antibodies block the growth of cancer cells by attaching themselves to HER2 and preventing the attachment of human epidermal growth factor to HER2 (175). According to a 2014 research by Luo et al., trastuzumab combined with chemotherapy dramatically enhanced OS and PFS in patients with advanced esophageal cancer, with an increased incidence of side events that were, for the most part, controllable (176). Subsequent studies have confirmed this result, and in 2023, JACOB found that the pertuzumab group had improved median OS and PFS values in patients with advanced metastatic gastroesophageal junction cancer compared to the control group, but unfortunately, there was an increased incidence of post-treatment adverse events (177). A 2019 Korean study comparing the difference in efficacy between chemotherapy alone, chemotherapy plus trastuzumab, and chemotherapy plus trastuzumab plus patuximab in gastroesophageal junction tumors showed that the latter two groups were significantly more effective than the first group, but unfortunately, this study did not document the probability of adverse events (178). In conclusion, anti-HER2 monoclonal antibodies are more effective than chemotherapy alone in prolonging patient life in advanced esophageal cancer and gastroesophageal junction tumors, but there is an urgent need to find ways to lower the incidence of side effects after treatment. The only literature on anti-VEGF receptor monoclonal antibodies is ramucirumab for the treatment of advanced esophageal cancer and gastroesophageal junction cancer. Ramucirumab in combination with paclitaxel is an effective second-line agent for the treatment of gastroesophageal junction tumors in Japan. Ramucirumab was found to potentiate the efficacy of the immunosuppressant nivolumab in the ATTRACTION-2 study, but the relationship between the two needs to be further investigated (179).

#### 3.3.2 Potential targets

In addition to several already known targets and corresponding monoclonal antibodies, an increasing number of studies are focusing on potential targets. For example, Zheng et al. extracted the monoclonal antibodies mAb-1E2 and mAb-2E3 against PAI-1, a cancer-associated fibroblast-derived PAI-1 known to promote the invasion and proliferation of esophageal cancer cells. This monoclonal antibody acted as an inhibitor of ESCC cell activity by blocking PAI-1 binding to LRP-1 and uPA. Moreover, PAI-1 plays a part in the recruitment of macrophages, indicating that this monoclonal antibody may be used in conjunction with immunotherapy and chemotherapy to prevent malignancies from evading the immune system (180).

Furthermore, an increasing number of researchers are turning their attention to the study of microRNA-, lncRNA-, and circRNA-related genes, which are likely to be potential targets for future targeted therapeutics. In 2022, a study found that circRUNX1, a circRNA related to the RUNX1 gene, could inhibit ESCC cell activity by hosting the microRNA-499b-5p fragment. This induction led to increased expression of the Treg surface protein FOXP3, which promotes the release of Treg-related cytokines and facilitates immune escape from ESCC. These findings suggest that circRUNX1 could be a potential target for targeted therapies in esophageal cancer (181).

Regarding miRNAs and lncRNAs, in addition to those mentioned above, miR-10527-5p (83), miRNA-21-5p (182), miRNA-142-3p (183), lncRNA SSTR5-AS1 (184), and lncRNA TPT1-AS1 (185) have been found to be closely associated with the metastasis and progression of esophageal cancer. These molecules could serve as effective targets for the treatment of metastatic esophageal cancer in the future.

It’s interesting to note that ferroptosis has two sides in tumor growth, necessitating careful target selection. Recently, it was found that DNAJB6 (186) and SCARA5 (187) can promote the process of ferroptosis in tumor cells and inhibit tumor progression.

## 4 Conclusion

In conclusion, our understanding of the mechanism of esophageal cancer metastasis is still limited. Current research on esophageal cancer mechanisms primarily focuses on cytokines that impact the stability of the tumor microenvironment and contribute to tumor immune escape, as well as RNA and its associated genes that mediate tumor metastasis. These studies provide potential targets for targeted therapy. Furthermore, we have summarized the progress in the comprehensive treatment of advanced and metastatic esophageal cancer, with a particular emphasis on targeted therapy and immunotherapy. Recent studies have explored the safety and efficacy differences between targeted therapy, immunotherapy, and chemotherapy in advanced esophageal cancer. However, there is a lack of trials investigating novel medications, but we anticipate that this will change in the future.

## Author contributions

WYS, YW: Writing - Original draft. WQY, YZ: Writing - Review and Editing. All authors have read and agreed to the published version of the manuscript.
